# Recent Advances in Application of Biosensors in Tissue Engineering

**DOI:** 10.1155/2014/307519

**Published:** 2014-08-06

**Authors:** Anwarul Hasan, Md Nurunnabi, Mahboob Morshed, Arghya Paul, Alessandro Polini, Tapas Kuila, Moustafa Al Hariri, Yong-kyu Lee, Ayad A. Jaffa

**Affiliations:** ^1^Biomedical Engineering and Department of Mechanical Engineering, Faculty of Engineering and Architecture, American University of Beirut, Beirut 1107 2020, Lebanon; ^2^Center for Biomedical Engineering, Department of Medicine, Brigham and Women's Hospital, Harvard Medical School, Cambridge, MA 02139, USA; ^3^Harvard-MIT Division of Health Sciences and Technology, Massachusetts Institute of Technology, Cambridge, MA 02139, USA; ^4^Department of Chemical and Biological Engineering, Korea National University of Transportation, 50 Daehak-ro, Chungju 380-702, Republic of Korea; ^5^Tissue Engineering Centre, Faculty of Medicine, National University of Malaysia (Universiti Kebangsaan Malaysia), 56000 Cheras, Kuala Lumpur, Malaysia; ^6^Department of Chemical and Petroleum Engineering, University of Kansas, Lawrence, KS 66045-7609, USA; ^7^Surface Engineering & Tribology Division, CSIR-Central Mechanical Engineering Research Institute, Mahatma Gandhi Avenue, Durgapur, West Bengal 713209, India; ^8^Department of Biochemistry and Molecular Genetics, Faculty of Medicine, American University of Beirut, Beirut 1107 2020, Lebanon

## Abstract

Biosensors research is a fast growing field in which tens of thousands of papers have been published over the years, and the industry is now worth billions of dollars. The biosensor products have found their applications in numerous industries including food and beverages, agricultural, environmental, medical diagnostics, and pharmaceutical industries and many more. Even though numerous biosensors have been developed for detection of proteins, peptides, enzymes, and numerous other biomolecules for diverse applications, their applications in tissue engineering have remained limited. In recent years, there has been a growing interest in application of novel biosensors in cell culture and tissue engineering, for example, real-time detection of small molecules such as glucose, lactose, and H_2_O_2_ as well as serum proteins of large molecular size, such as albumin and alpha-fetoprotein, and inflammatory cytokines, such as IFN-g and TNF-*α*. In this review, we provide an overview of the recent advancements in biosensors for tissue engineering applications.

## 1. Introduction

Biosensors have gained enormous attention in recent years in medicine and nanotechnology, and there is a growing interest in its application in tissue engineering. Since the development of the first oxygen biosensor by Lel and Clark in 1962 [1], researchers in diverse fields have developed numerous biosensors for applications in medicine, biotechnology, and defense against bioterrorism, as well as foods, beverages, and environmental and agricultural applications [2].

Recently, biosensors have shown immense potential for applications in tissue engineering and regenerative medicine. Both tissue engineering and regenerative medicine are rapidly growing fields in biomedical engineering presenting enormous potential for development of engineered tissue constructs for restoring the lost functions of diseased or damaged tissues and organs [3, 4]. Biosensors are gradually becoming an integral part of such tissue engineering systems particularly in microfluidic tissue engineering models as they can sense specific biological molecules within the miniaturized tissue constructs in real-time, at very low concentration levels, through ultrasensitive optical, electrochemical, or acoustic sensing systems. The most frequent use of biosensors so far has been in blood glucose monitoring [5, 6]. Enzymes, antibodies, and receptors have been widely used in biosensors as biological sensing elements [7]. Biosensors have also shown potential for* in vivo* sensing of disease-specific biomarkers [8]. The device in an* in vivo* environment can monitor real-time biological signals, such as the release of proteins or antibodies in response to tissue damage, muscular dystrophy, cardiac infarction, inflammatory events or infections. Thus biosensors possess a unique advantage to inform health-related complexities in a timely manner which is a powerful tool for early stage disease detection and treatment in clinical settings [9].

To precisely sense the biological signals in a cellular microenvironment, a probe with micro- or nano-dimensions is desirable. For this purpose, sensors with nanoscale dimensions, such as nanotubes or nanowires, have been developed for effective biosensing and diagnostics purposes. They can be used to measure pH or functionalized with specific capture molecules to identify very low quantities of biological and chemical species [9]. For example, nanocantilevers were used to monitor the serum protein marker levels and to determine the content of specific DNA moieties [10, 11]. Quantum dots, which are highly fluorescent semiconductor nanocrystals, can also be used to detect specific protein or DNA [12].

In fact, research is in progress to use nanobiosensors in combination with signaling and therapeutic delivery devices for* in vivo* screening and treatment [13–15]. Interestingly, biosensors with different micro- and nanostructured surfaces have been successfully used for both short-term and long-term* in vivo* studies [16]. The sensors were biocompatible and demonstrated increased biointegration, adhesion, proliferation, differentiation, and signaling potentials. To date, the application of biosensors in biomedical engineering is still limited and is at its early stage of development. Yet, the clinical potential can be realized. However, the combination of these two multidisciplinary technologies offers great promise for their eventual translation from bench to bed-side applications in the near future. The objective of this review is to present a comprehensive overview of the fundamental principles for biosensor design, fabrication, and operation mechanisms and to provide insights to their rapidly growing and future potentials in the field of biomedical engineering, particularly with respect to tissue engineering.

## 2. Fundamentals of Biosensors

### 2.1. Definition and Types of Biosensors

**Figure 1:**
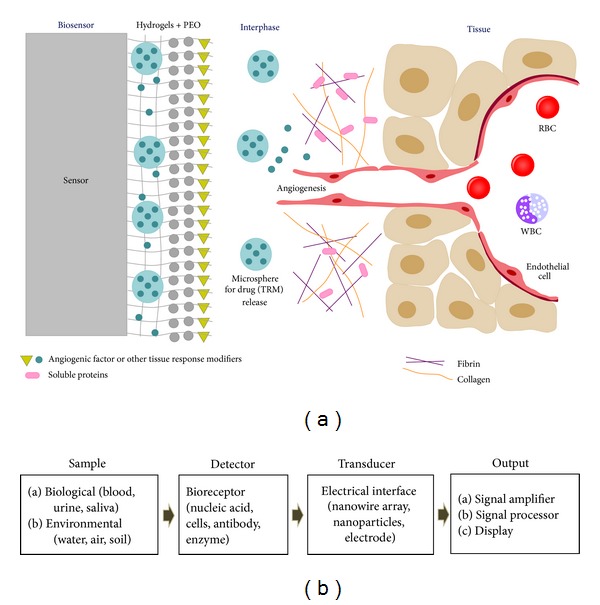
Schematic representation of the working principle of biosensors: (a) interaction between tissue, interphase, and biosensors. Figure 1 is reproduced with courtesy of http://www.tankonyvtar.hu/. (b) The components involved in biosensing.

A biosensor can be defined as “a self-contained analytical device that combines a biological component with a physicochemical component for the detection of an analyte of biological importance.” It is typically comprised of three fundamental components, such as (a) a detector to detect the stimulus, (b) a transducer to convert the stimulus to output signal, and (c) a signal processing system to process the output and present it in an appropriate form Figure 1.

Biosensors can be classified into different types either based on their sensing components or the transducer components as described below.

### 2.2. Bioreceptors or Biosensing Components

The biosensing components of biosensors can be divided into two types, namely, catalytic type and affinity type. The catalytic type sensors include enzymes, microbes, organelles, cells, or tissues, while the affinity type includes antibodies, receptors, and nucleic acids. Some of the important ones among these types are discussed below.

#### 2.2.1. Enzymes

The enzymes used as bioreceptor components in biosensors are usually proteins of oxidase type that can selectively react with specific analytes, consume dissolved O_2_, and produce H_2_O_2_ that is an easily detectable compound. Other mechanisms of enzyme based biosensing include the detection of enzyme activation or inhibition by the analyte and the modification of the enzyme properties by the analyte. The enzyme molecules can be directly immobilized on the transducer surfaces using entrapment in gels, attachment through covalent bonding, physical adsorption on the surfaces, or other available techniques [17, 18]. The advantages of enzyme based biosensing include the commercial availability of enzymes at high purity level, the high specificity of their binding capabilities, the suitability with various transduction techniques, and the ability to detect a wide range of analytes. Besides, since the action mechanism of enzymes is of catalytic nature where the enzyme itself remains unaltered at the end of the reaction, these sensors can be used continuously. The disadvantages of the enzyme based sensors include the limited stability of the enzymes and the dependency of their activities on various factors such as pH, ionic strength, chemical inhibition, and temperature.

#### 2.2.2. Microbes

The use of microbes has a number of advantages as biological sensing component in the production of biosensors. They are present all over and have a great capacity to acclimatize to undesirable conditions and to develop the ability to metabolize new molecules with time. Microbial cells are cheaper than enzymes or antibodies. They can carry out several complex reactions while maintaining their stability. Whole cells can be used either in a viable or nonviable form. Viable cells have gained importance in the manufacture of biosensors and these cells metabolize various organic compounds either anaerobically or aerobically resulting in various end products like ammonia, carbon dioxide, acids, and so forth that can be monitored using a variety of transducers. The use of microbial biosensors is common in environmental fields that include the detection of harmful bacteria or pesticides in air, water, or food and biological oxygen demand [19–21].

#### 2.2.3. Organelles

The compartments located inside the cells are known as organelles. Each of the organelles has individual functions such as lysosome, chloroplast, and mitochondria. Mitochondria are responsible for calcium metabolism and controlling the calcium dependent pathways in cells. Previous studies proved that presence of high concentration of calcium stimulates the mitochondria to open the calcium channels. This bioinspired strategy can be used to measure calcium concentration in medium. Application of mitochondria for water pollution detection is another application of organelles in biosensor [22].

#### 2.2.4. Cells and Tissues

Cells have been often used in bioreceptors because they have high sensitivity to adjacent environment. The attachment on the surface is the main characteristic of cells, so they can be easily immobilized. They are frequently used to detect global parameter like stress condition, toxicity, and organic derivatives and to monitor the treatment effect of drugs. Cells were also used in ion selective transducers [23, 24]. Tissues are also used in biosensors as they contain large quantity of enzymes. They offer a number of advantages over cells and organelles such as, easier immobilization, higher activity and stability, low price, and existence of necessary cofactors to function [25]. Their disadvantages include lack of specificity because of the presence of undesirable enzymes which can make the reaction complicated and result in ambiguous and less reliable outputs.

#### 2.2.5. Antibodies

Antibodies are proteins produced by B-Lymphocytes in response to antigenic stimulation. The antibody based sensors are also known as immunosensors. Usually, antibodies are used in surface plasmon resonance (SPR) biosensors to design target specific sensors for detecting specific biomolecules. This is a simple mechanism that works through antigen-antibody interaction process. The antibodies are usually linked to the surface of transducers through covalent bonds such as amide, ester, or thiol bonds. The transducer surface needs to be modified by polymers or monomers to introduce functional groups such as carboxyl, amino, aldehyde, or sulfhydryl groups to facilitate conjugation between the antibody and transducer. To date, many antibodies have been made available in the market and used in immunoassays. They are more accurate and faster compared to the traditional assays [26]. However, there are some limitations for antibody based biosensors, such as the irreversible interaction and the strength of the binding affinity. The latter is dependent on conditions such as pH and temperature, making the results highly variable due to the measurement conditions [27, 28].

#### 2.2.6. Nucleic Acids

Oligonucleotides integrate in nucleic acid biosensor with a signal transducer. Oligonucleotide probe is immobilized on the transducer to detect DNA/RNA fragments. The detection process is based on the code of complementary nucleotide base pairing, adenine (A): thymine (T) and cytosine (C): guanine (G) in DNA. The hybridization probes in the sensor can then base pair with the target sequences and create an optical signal [27].

### 2.3. Biotransducer Components

The transducer component of biosensors can be grouped into different types such as electrochemical, optical, acoustic, and calorimetric types.

#### 2.3.1. Electrochemical Biosensors

Electrochemical biosensors are mainly used for the detection of hybridized DNA, glucose concentration, and so forth. Electrochemical biosensors can be classified based on measurement of electrical parameters such as: (i) conductometric, (ii) amperometric, and (iii) potentiometric types. Electrochemical biosensors usually contain three electrodes: a reference electrode, a working electrode, and a counter electrode. The reaction for target analyte takes place on the active electrode surface. The reaction causes either electron transfer across the double layer or can contribute to the double layer potential. These kinds of biosensors are often made by screen printing the electrode patterns on a plastic substrate, coated with a conducting polymer and then some protein is attached. All biosensors usually involve minimal sample preparation as the biological sensing component is highly selective and the signal is produced by electrochemical and physical changes in the conducting polymer layer.

#### 2.3.2. Optical Biosensors

Optical biosensors are usually made based on optical diffraction. These sensors can detect microscopic changes when cells bind to receptors immobilized on the transducer surface. They use the changes in mass, concentration, or number of molecules to direct changes in characteristics of light. Researchers have used optical techniques such as SPR and ellipsometry for the detection of bacterial pathogens [29, 30].

#### 2.3.3. Other Biosensors: Acoustic, Thermometric, Magnetic and Piezoelectric Biosensors

The acoustic transducers used in biosensors are based on either the bulk acoustic wave or the surface acoustic wave. The transduction is through detection of changes in their physiochemical properties, such as mass density, elasticity, viscoelasticity, or electrical conductivity [31]. Calorimetric transducers, on the other hand, depend on changes in the temperature of the sensing site due to biochemical reactions [32]. The thermometric, magnetic, and piezoelectric transducers have failed so far to have any practical impact on tissue engineering applications [33].

## 3. Applications of Biosensors in Tissue Engineering

Biosensors can be of immense importance in tissue engineering applications, particularly in maintaining three-dimensional cell cultures [34] and developing “organs-on-chips” models, where concentrations of biomolecules such as glucose, adenosines, and hydrogen peroxide levels play important roles in determining the fate of the cells and tissues. Living cells are well known to transmit various physical and chemical signals, such as changes in consumption of oxygen, pH, membrane potentials, ion concentrations, and release of various metabolic compounds and proteins [35]. Monitoring these analytes can give insights into cellular activities in real time.

### 3.1. Detection of Small Molecules

#### 3.1.1. Glucose

In clinical applications, biosensor-based monitoring of blood glucose concentration has now become a major diagnostic method to accurately trace diabetes with high levels of glycated hemoglobin (HbA1c). In tissue engineering applications, however, continuous monitoring of glucose in culture media is used as an indicator of metabolic activities of cells [33, 36]. A number of different biosensing approaches have been provided for glucose monitoring. This includes electrochemical biosensors that are frequently used for glucose oxidase or glucose dehydrogenase detection from blood to interstitial fluids [37]. Several studies have reported optical biosensors for glucose detection using inactive apoenzymes, binding proteins, and receptors. This includes alternative strategies and approaches for development of reversible, implantable, and/or in-line sensing systems [38–40]. Investigations are also focused on discovering techniques to measure the glucose content noninvasively. Despite some promising improvements in the technology in recent years, there is still no noninvasive tool that is in use in clinical practice [41]. In the contrary, polarimetry [42, 43], diffuse reflection spectroscopy [44, 45], absorption/transmission spectroscopy [46, 47], thermal emission spectroscopy [48, 49], photoacoustic spectroscopy [50], and near-infrared spectroscopy (NIRS) [51–53] have demonstrated promising successes in measuring blood glucose levels with high accuracy. The responsiveness of these methods, however, is considerably slow due to the weak glucose absorption bands (combination bands) and the presence of various undesired bands from other constituents of the system. On the other hand, the mid-infrared (MIR) region involves a prominent glucose absorption band and it gives isolated band in human blood [54–57]. However, MIR method is limited by strong water absorption and background fluctuation that frequently hamper the results. Photoacoustic [50] and thermal radiation methods [47, 49] also demonstrate variable results for water accumulation. Recently a noninvasive and noncontacting technique, the wavelength modulated differential laser photothermal radiometry (WM-DPTR), has been developed for continuous or intermittent glucose monitoring in the MIR range. This can be applied to measure serum-glucose levels in human skin* in vitro* [58, 59]. These recent advances in application of nanobiosensor technologies in monitoring of glucose concentrations are primarily targeted toward the measurement of blood glucose level in diabetic patients [60]. These techniques can be equally applied to monitor the cellular metabolism in engineered tissue constructs in real time during their fabrication, proliferation, and growth, given that consumption of glucose by the cells is the best indication of cell metabolism [61].

#### 3.1.2. Hydrogen Peroxide (H_2_O_2_)

Accurate and reliable measurement of H_2_O_2_ is of paramount importance in both tissue engineering and clinical applications. The monitoring of H_2_O_2_ allows detecting the presence of oxidative stress or hypoxic conditions in the cell and tissue culture. Currently, available analytical methods of H_2_O_2_ measurement include techniques such as electrochemistry, photometry, and titration [62]. High (usually ≥50 *μ*M) levels of H_2_O_2_ are cytotoxic to human and to a wide range of animal, plant, and bacterial cells. Abnormal level of H_2_O_2_ is highly detrimental to the biological systems. In tissue engineering applications, fluorescence-based and electrochemical methods have been widely used for H_2_O_2_ detection. However, these techniques have limitations including poor H_2_O_2_ specificity, low sensitivity, difficulty in applying to the biological environments, and invasiveness of measurement (e.g., electrode-based method). Amperometric enzyme based biosensors have gained much attention due to their relatively high expediency, selectivity, and sensitivity [63, 64]. Development of sensitive and steady sensors stems from the efficient binding of enzyme to solid electrode surface [65]. Numerous strategies have been developed to efficiently immobilize enzymes on the electrode surface for H_2_O_2_ detection. These include, but are not limited to, polymers [66, 67], quantum dots [68], and various nanomaterials [69–71]. Among these, the nanomaterials based methods have been the most widely explored ones for this purpose. For example, electrochemical biosensors based on silver (Ag) nanoparticles (AgNPs) can be used as an important component for the electrode. Xu et al. [72] developed a H_2_O_2_ biosensor based on the direct electrochemistry of hemoglobin (Hb) in Hb-Ag sol on glassy carbon (GC) electrode. Hb showed a pair of distinct redox peaks on GC electrode and exhibited high sensibility, good reproducibility, and long-term stability. Nanoprobes, as a part of nanobiosensors, are able to detect H_2_O_2_ using detection principles (chemiluminescence, fluorescence, localized surface plasmon resonance, near-infrared absorption, and electrochemical methods), materials of nanoparticle matrix, and dependence on enzymes [73–76]. The use of nanoprobes/sensors in H_2_O_2_ detection has certain advantages in both* ex-vivo* and* in-vivo* tissue engineering. In general, nanoprobes are in the size range of 10–500 nm, which is much smaller than the size of biological cells and this minimizes physical disturbance to cells or tissues while performing measurements. Besides, nanoprobes offer multifunctionalities and they can be made tissue or cell specific by conjugating target-specific ligand moieties onto the nanoparticle decorated electrode surface.

#### 3.1.3. Adenosines

Extracellular adenosine diphosphate (ADP) and adenosine triphosphate (ATP) are vital multifunctional molecules present in blood, heart, and liver. Apart from its main role in cellular metabolism, ATP is now recognized as an important extracellular signaling agent. It can modulate a number of physiological pathways by activating specific plasma membrane receptors. Luciferase-based methods have long been used for measuring adenosines; however, they have limited application* in vivo* due to their low sensitivity and resolution. Consequently, there is a need for alternative and more convenient methods for ATP measurement. Biosensor can offer an option for* in situ* extracellular ATP measurement and sensitive* in vivo* applications [77, 78]. It is known that the extracellular ATP, ADP, and uridine triphosphate (UTP) are involved in a wide variety of different biological responses such as apoptosis, cell proliferation, migration and differentiation, cytokine release, and necrosis [79]. Nucleotide signaling participates in several critical physiological and pathological events such as immune system maturation, neurodegeneration, inflammation, and cancer [80]. The* in vivo* extracellular ATP concentration can increase to reach the hundred micromolar level in many diseases such as hypoxia, trauma, ischemia, cancer, or inflammation [81]. In general, extracellular ATP is measured in the cell supernatant by using the standard bioluminescence luciferin/luciferase assay. However, this method does not permit real-time measurement of the extracellular ATP concentration. Llaudet and coworkers developed a microelectrode recording system for* in vivo* measurements of ATP [77]; however, their method requires the electrode to be placed inside the tissue which may affect the ATP measurement. Another group used a microelectrode biosensor to measure the purine in granule primary cell culture for neuronal regeneration [82]. Schneider and coworkers engineered a scanning tip, coated with the ATPase-containing S1 myosin fragment, to identify the sources of ATP release and to measure the ATP concentration [83]. However, this method is complicated for clinical applications. Hayashi and coworkers developed a biosensor [84], which can be placed near the ATP-releasing target cells. This technique gives an accurate result of the extracellular ATP concentration. Recently, Xie and coworkers developed a novel localized surface Plasmon resonance (LSPR) array chip for facile, label-free, and high throughput detection of ATP using a normal microplate reader. The report suggested that the developed LSPR sensor chip can be used for miniaturized and high throughput detection of biological samples in tissue engineering applications [85].

### 3.2. Detection of Functional Protein Molecules

It is important to measure the activities of functional protein molecules such as bioenzymes released from cells, under different microenvironmental conditions to understand the fundamentals of cell biology for therapeutic, diagnostic, and tissue engineering applications. Matrix metalloproteinase (MMPs), a member of proteinases family, is released by cells as a biological response to their natural tissue remodeling processes [86]. MMPs are also released to various extents in response to different pathological conditions including cancer [87]. Thus MMP proteins can act as biomarkers for different diseased states which can be detected and quantified with the help of biosensors. At present, colorimetric methods with commercially available proteinase assay kits are used to measure proteinase activity [88]. Enzyme-responsive polymers are also popular as sensing elements in biological devices [89–91]. In these cases, fluorescent molecules are connected to a quencher through peptide sequences and cleavage of the peptide sequences by proteinase enzymes gives a fluorescence signal which can be quantified to monitor the target protein concentration and activities [92]. Nevertheless, sometime the labeling method is not suitable, especially in detecting suboptimal biomolecule levels in 3D* in vitro* tissue culture conditions. Recently, label-free biosensors have been developed based on sensitive optical biosensing methods [93, 94]. Such biosensors exhibit real-time monitoring of abnormalities of extracellular proteins, such as autophagy and proteostasis [95, 96] as well as* in vitro* cell culture conditions [97]. Biophotonics based biosensors can also be used to monitor the level of extracellular proteins, hormones, and soluble molecules.

Biosensors have been used for early detection of cancer biomarkers from blood samples in a noninvasive manner. Surface plasmon resonance (SPR) and electrochemical biosensors have been successfully used for the detection of carcinoembryonic antigen (CEA) biomarkers for early diagnosis of lung cancer in serum [98–100]. Lab-on-chip and optical biosensors have been used for detection of epidermal growth factor receptor (EGFR) biomarker for early diagnosis of cancer [101, 102]. In engineered tumor models, fluorescence based biosensors represent useful tools for the early detection of biomarkers in clinical diagnostics, for monitoring disease progression and response to treatment/therapeutics [103–105]. Protein kinases are major proteins in cell signaling pathways and disease progression and can act as real-time biomarkers in response to different therapeutics which can also be detected using biosensors [106–108]. The normal ranges of many biomarkers are at nanogram and picogram levels and these trace amounts can only be detected via highly sensitive biosensing systems with proper surface chemistries, nanomaterials functionalization, and signal amplification methodologies. On the other hand, there are still many obstacles for determination of disease markers using biosensors like the reflection between the sensing molecule and the target, nonspecific binding in the case of serum or real patient samples, the small size of the target, and the effect of the microfluidics systems of the sensors on the measurement process. These issues are the major challenges in biosensor systems and they need more investigation to be overcome.

### 3.3. Detection of Other Analytes

Different types of biosensors have been developed for detection of pathogenic microbes. Amperometric biosensors have been developed for indirect detection of* E. coli* and direct detection of Salmonella [109, 110]. Members of the Enterobacteriaceae family can be detected by Piezoelectric immunosensors [111].* Neisseria meningitidis and Brucella melitensis* are detectable by a light addressable potentiometric sensor [112]. Endotoxins are complex lipopolysaccharides (LPS) of outer cell wall of all Gram-negative bacteria causing fever, multiorgan failure, septic shock, sepsis, meningococcemia, and severe morbidities like neurologic disability and hearing loss. It is essential to detect endotoxin for quality control in biological products, recombinant therapeutic products, medical devices, serological products, food, and water security [29, 113].* E. coli* endotoxin was detected by biosensor using fluorescence technique, where the lower limit of detection was 10 ng/mL and detection time was 30 seconds [114]. Endotoxin from* Salmonella minnesota* was identified at 0.1 ng/mL level using an amperometric biosensor [115] and 0.1 pg/mL level by a piezoelectric biosensor [116]. The recognition and quantitation of viruses are essential for a broad variety of applications from sanitation and food production to diagnostics and therapeutics [117, 118]. Dengue virus has been effectively detected using optical biosensor [119], human immune deficiency virus (HIV) by SPR EIS biosensor [120, 121], and liver inflammation caused by Hepatitis C virus by optical and quartz crystal microbalance (QCM) biosensor [122, 123].

## 4. Recent Trends in Biosensors

### 4.1. Quantum Dots Based Optical Biosensors

**Figure 2:**
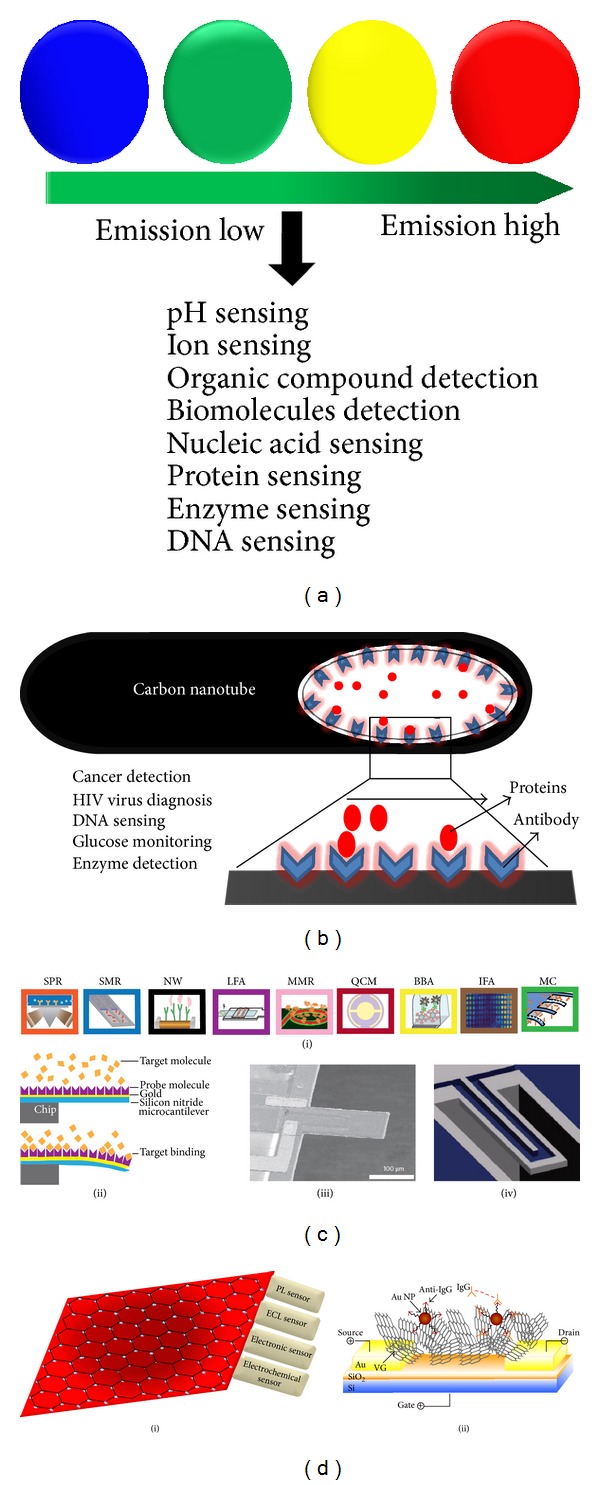
Schematics for some recent advancement in biosensors applicable in tissue engineering. (a) Variation of color in quantum dots (blue, green, yellow, and red) based on their emission wavelength. (b) Carbon nanotube based biosensor for detecting various cell secreted biomolecules from tiny amount of sample. (c) Some MEMS based biosensors: (i) SPR: surface-plasmon resonance; SMR: suspended microchannel resonator; NW: nanowire; LFA: lateral flow assay; MRR: microring resonator; QCM: quartz crystal microbalance; BBA: biobarcode amplification assay; IFA: immunofluorescent assay; MC: microcantilever. (ii) static-mode surface-stress sensing by a MEMS device (iii) scanning electron micrograph of dynamic mode MEMS device and (iv) suspended microchannel resonator (SMR). (d) (i) Graphene and its derivatives (graphene oxide, graphene quantum dots) based sensors. (ii) Vertically-oriented graphene based field effect transistor-sensor by direct growth of VG between the drain and the source electrodes. (c) and (d) (ii) reproduced from [13] and [132], respectively, with permission from Nature Publishing Group.

Semiconductor quantum dots (Qdots) are one of the most promising optical imaging agents for* in vitro* (biosensors and chemical sensors) and* in vivo* (noninvasive imaging of deep tissues) diagnosis of diseases due to their ultra-stability and excellent quantum confinement effects [1, 7, 124, 125]. Qdots have a broad excitation and narrow size (5–10 nm in diameter) tunable emission spectrum with narrow emission band width. These unique properties facilitate the use of Qdots in a wide range of fields such as biology, biosensor, electronics, and solar cells [5–7]. The surface modification and decoration of Qdots have inspired the development of novel multimodal probes based biosensors through linking with peptides, nucleic acids, or targeting ligands. Since the fluorescence intensity of Qdots is highly stable and sensitive, fluorescence transduction based on chemical or physical interaction occurs on the surface either through direct photoluminescent activation or through quenching [9, 10]. Qdots have been widely investigated for possibilities of sensing pH, ions, organic compounds, and biomolecules (nucleic acids, protein, and enzymes), as well as other molecules of biological interests (Figure 2(a)) [11, 12, 15, 16]. While the toxic effects of some Qdots have still remained as a concern [14], the recent advancements in application of Qdots in tissue engineering to detect the enzyme and biomolecules are significant achievements of biosensing research.

### 4.2. Carbon Nanotube Based Biosensors

The unique chemical and physical properties of carbon nanotubes have introduced many new and improved sensing devices. Early cancer detection in* in vitro* systems is one of the most recent, attractive, and breakthrough inventions from carbon nanotube based biosensors [126, 127]. The specific antibody coated surface of carbon nanotubes could be used for detecting proteins and viruses of interest (Figure 2(b)). The key insights of this invention are noticeable changes in the electrical conductivity of the nanotubes when the distance between the antibody and protein changes. The change of distance can be detected by an electrical meter. Carbon nanotubes have been widely investigated for promising applications in dehydrogenase, peroxidase and catalase, DNA, glucose, and enzyme sensors [128]. Carbon nanotube-based electrochemical transduction demonstrates substantial improvements in the activity of amperometric enzyme electrodes, immunosensors, and nucleic-acid sensing biosensors [129]. The enhanced performance and properties of carbon nanotubes can thus be important in tissue engineering to overcome the current limitations such as improving elasticity, flexibility, cell growth, and cell patterning.

### 4.3. MEMS/NEMS Based Biosensors

The growing need for miniaturization of biosensors has resulted in increased interests in microelectromechanical systems (MEMS) [130, 131], nanoelectromechanical systems (NEMS), and microfluidic or lab-on-a-chip systems based biosensors [13, 132]. Such miniaturized systems offer more accurate, specific, sensitive, cost-effective, and high performance biosensor devices [133]. The different methods that have been used in MEMS based biosensors include optical, mechanical, magnetic, and electrochemical detections (Figure 2(c)). Organic dyes, semiconductor quantum dots, and other optical fluorescence probes have been used in optical detection methods [134], while conjugation of magnetic, paramagnetic or ferromagnetic nanoparticles has been used in magnetic MEMS biosensors [135]. Mechanical MEMS biosensors are designed based on one of the two factors, namely, changes in surface stress and changes in mass. Biochemical reaction and adsorption of analytes on the cantilever result in changes of surface stress. The electrochemical MEMS based biosensors use amperometric, potentiometric, or conductometric detection.

### 4.4. Graphene Based Biosensors

Graphene based biosensors have attracted significant scientific and technological interests due to the outstanding characteristics of graphene, such as low production cost, large specific surface area, good biocompatibility, high electrical conductivity, and excellent electrochemical stability [136–138]. The 2D structure of graphene favors *π*-electron conjugation and makes its surface available to other chemical species. Therefore, graphene is emerging as a preferred choice for the fabrication of various biosensor devices in tissue engineering [136, 139, 140].

#### 4.4.1. Graphene Quantum Dots Based Biosensors

Graphene derivatives, especially 0D graphene quantum dots (Gdots), are photoluminescent materials derived from graphene or carbon fibers [141–143]. Gdots possess very unique optical properties in combination of quantum confinement and zig-zag edge effects. The ultra nanosized Gdots with wide range of excitation/emission spectrum are promising candidates for applications in electronic, photoluminescence, electrochemical and electrochemiluminescence sensors fabrication for various chemical and biological analyses [144]; see Figure 2(d). Gdots are superior compared to other well-known optical imaging agents such as organic dyes and cadmium based Qdots due to their high photostability against photobleaching, blinking, biocompatibility, and low toxicity [145, 146]. These unique properties enable the Gdots to be used in electronic sensors, electrochemiluminescence sensors, electrochemical sensors, and photoluminescence sensors [147]. In electronic sensors, Gdots are mainly used in single electron transistor based charge sensors, unlike the extensive application of graphene in field effect transistors. Several methodologies have been reported for synthesis of blue, green, yellow, and red Gdots from graphene or carbon fiber [141–143]. The colors of Gdots are related to the basic factors such as size, shape, excitation, pH, band gap, degree of oxidation, surface functionalization, and doping of S and N. The as-synthesized Gdots are very convenient for detecting any positively charged ions (cationic) such as Ag^2+^ and Fe^3+^ through charge-to-charge interactions [148]. The tunable size of Gdots can be used for ssDNA detection, enzyme immobilization, and avian leukosis virus subgroup J (ALVs-J) detection. Decoration of Gold (Au) on the planer surface of Gdots offers a wide range and low detection limit for detection of H_2_O_2_. A Gdot based electrochemiluminescence sensor was investigated for detecting Cd^2+^, cysteine, and ATP. Low cytotoxicity, low cost, excellent solubility, and ease of labeling of Gdots are also attractive for application in development of novel ECL biosensors.

#### 4.4.2. Graphene Based Glucose Biosensor

The glucose biosensors, as mentioned in earlier section, can be used in tissue engineering for continuous measurements of metabolic activities of cells. Graphene oxide (GO), the precursor material of graphene, has been used as a novel highly efficient enzyme electrode for the detection of glucose in phosphate buffer saline solution (PBS) [149, 150]. The amine functional groups of glucose oxidase (GOD) were covalently attached to the carboxyl functional groups of GO. The direct electrochemistry of GOD immobilized with the chemically derived graphene was investigated in detail [151, 152]. The GOD immobilized electrode retained its native structure and catalytic activity with effective direct electron transfer reaction rate constant. The electrocatalytic activity of the chemically derived graphene sheets exhibited enhanced electrocatalytic activity towards the detection of glucose in PBS. The designed electrodes displayed excellent sensitivity, selectivity, and reproducibility, suggesting their possible use in the fabrication of low cost glucose sensing devices.

Graphene-based nanocomposite materials have been extensively used in the fabrication of glucose biosensor [153–158]. Graphene/gold nanoparticle (AuNP)/Nafion nanocomposite biosensor showed typical catalytic oxidation response to glucose and the response was very fast upon the addition of glucose [154]. The sensing efficiency and detection limit of the graphene-based glucose biosensors were found to increase when silver nanoparticle (AgNP)/AuNP hybrid was used to catalyze electrochemical reaction of GOD [155].

**Figure 3:**
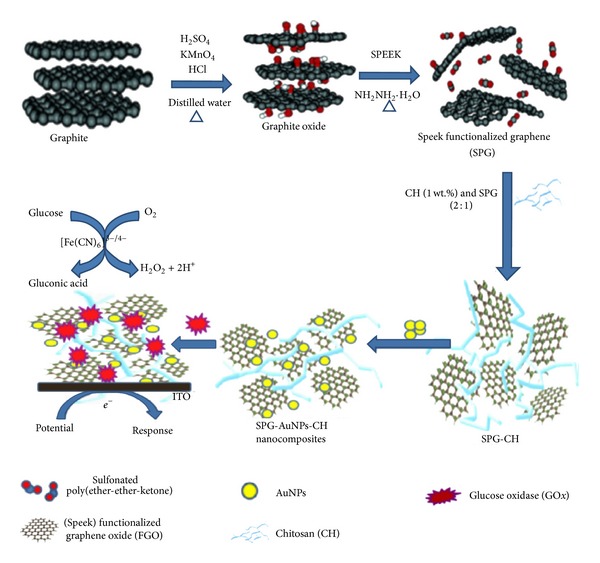
Schematic illustration for the preparation of SPEEK functionalized graphene and the biochemical reaction mechanism of the immobilized GOD toward glucose. Figure 3 is reproduced with permission from Elsevier [159].

**Figure 4:**
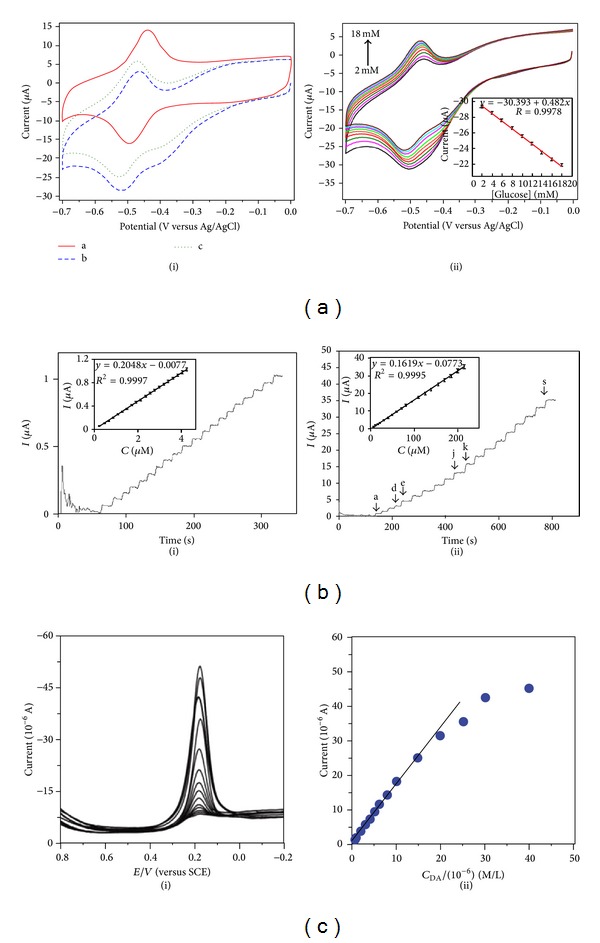
Some representative experimental data from graphene based biosensors. (a) Graphene based glucose biosensor: (i) O_2_ saturated PBS solution without glucose and (ii) O_2_ saturated PBS solution with different concentrations of glucose. (b) Graphene based cholesterol biosensor: (i) 0.25 *μ*M cholesterol and (ii) (a-dd) 5 *μ*M, (e-j) 10 *μ*M, and (k-s) 15 *μ*M cholesterol. (c) EDTA-RG/Nafion electrode. (i) Concentrations from 0.20 to 40.00*μ*M (with 1 mM AA in pH) 7.2 PBS. (ii) The relation between the current and concentrations (figures reproduced from [153, 160, 168] with permission from Elsevier and American Chemical Society, respectively).

The long-term stability of the developed biosensor was examined over 30 days and was found to be stable after the immobilization of the electrode with GOD. Wu et al. [156] showed that platinum nanoparticle (PtNP) decorated graphene/chitosan nanocomposite film could be used for the detection of glucose. The biosensor showed a wide linear range with fast response and high sensitivity. However, the standard deviation and detection limit of the graphene/chitosan nanocomposite biosensor were found to decrease in absence of PtNP as reported by Kang et al. [157]. Ionic liquid modified graphene electrodes exhibited excellent response time, <5 s, and good sensitivity [158]. Ionic liquid functionalized graphene-based glucose sensor retained its sensitivity and selectivity after immersion in PBS at low temperature for a few weeks. Liang et al. [153] showed the glucose sensing efficiency of the electrochemically reduced carboxyl graphene. The designed biosensor showed linear response to glucose at moderate concentrations with a detection limit of 0.02 mM. Therefore, it is seen that the graphene-based glucose sensors are highly sensitive, selective, and reproducible in nature. The schematic for a glucose biosensor using sulfonated poly-ether-ether-ketone (SPEEK) modified graphene is shown in Figure 3 [159]. The sensor can successfully detect glucose (Figure 4(a)) both in absence and in presence of commonly existing interfering species such as ascorbic acid (AA), uric acid (UA), and dopamine.

#### 4.4.3. Graphene-Based Cholesterol Biosensor

Cholesterol and its esters are the essential components found in the cell membranes of all human and animal cells. The normal cholesterol limit in human serum is in the range of 1.0–2.2 mM and its excessive accumulation in blood results in fatal diseases. Gholivand and Khodadadian [160] prepared cholesterol biosensor using graphene/ionic liquid modified glassy carbon electrode. Cholesterol oxidase (ChOx) and catalase (CAT) were immobilized to develop highly sensitive amperometric cholesterol biosensor. The RSD was found to be <5% and it showed good reproducibility with minimal interference from AA and UA (Figure 4(b)). The effect of PtNP on the biosensing efficiency of cholesterol based on graphene electrode was also investigated in detail [161]. The detection limit was found to be 0.5 nM in absence of ChOx or cholesterol esterase. These types of biosensors exhibited excellent sensitivity and linear response for the detection of cholesterol in physiological solutions (Figure 4(b)). The use of this kind of biosensor for the detection of free cholesterol exhibited great promise for the use in* in vitro* measurements.

#### 4.4.4. Graphene-Based Hydrogen Peroxide Biosensor

A novel H_2_O_2_ biosensor was fabricated using graphene/Fe_3_O_4_-AuNP and graphene/Fe_3_O_4_-AuNP nanocomposites coated with horseradish peroxidase [162, 163]. The biosensor showed excellent performance towards the detection of H_2_O_2_. The linear response of the biosensor was in the range of 2.0 × 10^−5^ moles lit^−1^ to 2.5 × 10^−3^ moles lit^−1^ with a detection limit of 1.2 × 10^−5 ^mol lit^−1^. The biosensor was highly sensitive, disposable, low cost, and strong anti-interference suggesting its utility as a reliable device for the detection of H_2_O_2_.

The sensitivity, selectivity, and detection limit of the enzymatic electrode are impressive. However, these electrodes suffer from the limitation of reproducibility, high cost, and complexity in enzyme immobilization procedure. The enzymatic electrodes are also very sensitive towards the change in pH of the solution, temperature, and toxic chemicals. In order to overcome these limitations, the use of nonenzymatic biosensors in the detection of biomolecules has been introduced as discussed below.

#### 4.4.5. Nonenzymatic Biosensors

Nonenzymatic detection of biomolecules using graphene-based electrodes has attracted significant attention due to its low fabrication cost, high sensitivity, and long-term stability. Li et al. [164] showed nonenzymatic detection of H_2_O_2_ using GO/MnO_2_ nanocomposites. The biosensor showed a linear range of 5–600 *μ*M with a detection limit of 0.8 *μ*M. The biosensor remained unaffected by the common interfering chemical species such as SO_4_^−2^, Cl^−^, NO_3_^−^, CO_3_^−2^, and citric acid. Wang et al. [165] showed that chemically derived graphene can detect dopamine with a linear range from 5 to 200 *μ*M in a large excess of AA. The nanocomposite of chemically derived graphene/chitosan/AuNP exhibited enhanced sensitivity towards the detection of DA and UA [166]. Zhong et al. [167] showed the effect of AgNP on the graphene thin films for the nonenzymatic detection of H_2_O_2_. The biosensor exhibited fast amperometric response time of <2 s and a good linear range with a detection limit of 3 × 10^−6^ M. A new type of chemically modified graphene was used for the selective detection of dopamine [168]. The surface of graphene was prepared by the silanization of graphene with N-(trimethoxysilylpropyl) ethylenediamine triacetic acid (EDTA-silane). Electrochemical detection of dihydronicotinamide adenine dinucleotide (NADH) based on graphene modified glassy carbon (GC) electrode was explored by Zhu et al. [169]. The sensor exhibited very high sensitivity due to the large amount of graphitic edges and porous structure of graphene sheets. The detection limit of the sensor was found to be 0.23 *μ*M with a RSD of 3.4% (Figure 4(c)).

DNA biosensor was fabricated using graphene/PANI nanocomposite films [170]. The synergistic effect of graphene and PANI improved the response of the electrode due to fast electron transfer at the electrode surfaces. Gupta et al. developed a highly sensitive and selective DNA biosensor using GO/AuNP composites [171]. The immobilization of the ss-DNA was confirmed from electrochemical impedance spectroscopy (EIS) analysis. The charge transfer resistance was found to increase with increasing the concentration of DNA. In comparison to the graphene/PANI biosensor, the detection limit and linear range were significantly improved in the GO/AuNP biosensor [172]. Huang et al. demonstrated a novel electrochemical biosensor for the selective and sensitive detection of DNA using polydopamine graphene composite [173]. The detection limit and the linear range of the biosensor have been found to be 3.2 × 10^−15 ^M and 1.0 × 10^−13^ to 1.0 × 10^−8 ^M, respectively. It also displayed high selectivity to differentiate one-base mismatched DNA. Therefore, it is seen that the graphene-based sensing system is facile, rapid, and cost-effective for the selective detection of various DNA.

### 4.5. Microfluidics and Biosensors

In the development of tissue engineered constructs, the need for reliable and sensitive tools to assess the artificial tissue environment has become vitally important. Such platforms require to be constantly monitored in terms of various physiologically relevant parameters to evaluate the functionality of the engineered tissue constructs. Microfluidic systems are able to mimic various signals that direct cell fate to create specific organ constructs by precise control of the chemical and mechanical stimuli at microscale [174]. Besides, microfluidic platforms allow handling tiny volumes of fluids (from microliters to nanoliters) in a high throughput automated manner and integrate several functions (e.g., multiplexing capability or possibility to carry out numerous reactions) in a single portable device [175, 176]. All these factors have made the microfluidic platforms highly attractive for tissue engineering particularly for organs on chips applications or for* in vitro* tissue models. In this context, the progress made towards the development of biosensors for point-of-care (POC) applications over the recent years can be highly beneficial for the tissue engineering applications as well. POC devices are analytical tools routinely used in clinical laboratories as well as at the patient bedside and are intended to provide a reliable response in a short time [177]. In recent years, their range of application has been further expanded by exploiting microfluidic technologies. Combining the POC capabilities with microfluidic platforms is the key challenge for researchers in tissue engineering, as many biomarkers have to be monitored to assess the functionality of any tissue engineered construct* in vitro*.

Recently, a number of studies have been reported on the combination of biosensor capabilities in microfluidic devices for tissue engineering. Weltin et al. presented a multiparametric microphysiometry platform to monitor the metabolism of T98G human brain cancer cells cultured in dynamic flow conditions [178]. A glass-made microfluidic device was employed to facilitate optical imaging and several microfabricated biosensors were integrated in the cell chamber as well as in upstream and downstream compartments. The levels of pH, oxygen consumption, and the production of cell metabolites (lactate and glucose) were monitored by using external equipment (e.g., potentiostat). Similarly, Hu et al. included a light-addressable potentiometric sensor (LAPS) in a microfluidic system to monitor the metabolism of human breast cancer cells in real time [179]. Microheaters and micropumps were also integrated to these systems for controlling the temperature and handling different fluids [180]. Moreover, aiming to study the behavior of adherent cells in tissue engineered constructs, impedance analysis is often used. Cells were cultured on the surface of a microfabricated electrode and were exposed to low-magnitude AC voltage. The electrical impedance measured in the system was correlated to various cell parameters such as number, type, state, and migration of cells. This technique is reported successfully in lab-made microfluidic systems [181–183] as well as in commercially available tissue culture plates (e.g., ECIS Cultureware Disposable Electrode Arrays from Applied BioPhysics Inc.).

A rapid translation of successful biosensing technologies to tissue engineering platforms is only at an early stage due to several challenges that researchers have to face for a proper integration in microfluidic systems [184]. Cell culture media include a very complex mixture of molecules (small molecules as well as complex molecules such as proteins and nucleic acids), extremely different in size and concentration. As a result, any biosensor has to be very accurate and sensitive to detect the analyte among a huge population of molecules. Considering the cell number into these systems, usually extremely low when compared to static conventional standard culture, the analytes can be produced at a very low level. In addition, other problems can occur such as surface biofouling at the biosensor level and nonspecific adsorption of target biomolecules in compartments different from the biosensor surface, leading to false response errors and decreased sensitivity. Strategies like the use of PEG of bovine serum albumin to passivate the surface of the microfluidic circuit can affect the system (i.e., the tissue engineered construct) in an unpredictable way and might be avoided.

On one hand, POC systems often present several capabilities in a single device, such as fluid handling, sample preparation (concentration, washing, etc.), and the possibility to perform different reactions for biochemical assays. On the other hand, the miniaturization and integration in microfluidic cell culture systems of these capabilities can be very challenging from a fabrication and operational point of view, although several attempts are reported [185, 186]. Most of these systems are employed as end-point detection tools where the sample is discarded after test. Thus a big challenge is their use in real time with no sample volume wasted, in order to perform several tests with the same (very small) amount of liquid.

## 5. Concluding Remarks and Future Directions

There has been a growing interest in biosensor research for applications in tissue engineering. However, the progress has remained limited. Even though numerous optical, electrochemical, magnetic, acoustic, thermometric, and piezoelectric sensors have been reported in the literature and, often, are already available in the market, showing great sensitivity and sensibility, the most successful among them in tissue engineering applications have been the electrochemical and optical ones, while the thermometric and magnetic transductions have failed to have any practical impact. The challenges for widespread applications of biosensors in tissue engineering include their miniaturization and integration in microfluidic systems. The continuous real time monitoring of analytes in tissue engineering is still at an early stage and can bring enormous possibilities in the field. The creation of microfluidic tissue engineering platforms with automated, sensitive, and real-time monitoring capabilities will hugely benefit the translation of such systems to clinics, as the full assessment of their parameters is a must for clinical applications. The successful widespread application of biosensors in tissue engineering particularly on microfluidic platforms will require standardization of the systems and the processes.
